# Stages and Conformations of the Tau Repeat Domain during Aggregation and Its Effect on Neuronal Toxicity*

**DOI:** 10.1074/jbc.M114.554725

**Published:** 2014-05-13

**Authors:** Satish Kumar, Katharina Tepper, Senthilvelrajan Kaniyappan, Jacek Biernat, Susanne Wegmann, Eva-Maria Mandelkow, Daniel J. Müller, Eckhard Mandelkow

**Affiliations:** From the ‡ German Center for Neurodegenerative Diseases (DZNE), 53175 Bonn, Germany,; the ¶ Center of Advanced European Studies and Research (CAESAR), 53175 Bonn, Germany,; the § Max Planck Institute for Neurological Research, Hamburg Outstation, c/o DESY, 22607 Hamburg, Germany, and; the ‖ Department of Biosystems Science and Engineering, Eidgenössische Technische Hochschule Zürich, Basel, 4058 Basel, Switzerland

**Keywords:** Aggregation, Neuron, Phosphorylation, Synapse, Tau Protein (Tau), Oligomers, Time-correlated Single Photon Counting, Toxicity

## Abstract

Several neurodegenerative diseases are characterized by the aggregation and posttranslational modifications of Tau protein. Its “repeat domain” (TauRD) is mainly responsible for the aggregation properties, and oligomeric forms are thought to dominate the toxic effects of Tau. Here we investigated the conformational transitions of this domain during oligomerization and aggregation in different states of β-propensity and pseudo-phosphorylation, using several complementary imaging and spectroscopic methods. Although the repeat domain generally aggregates more readily than full-length Tau, its aggregation was greatly slowed down by phosphorylation or pseudo-phosphorylation at the K*X*GS motifs, concomitant with an extended phase of oligomerization. Analogous effects were observed with pro-aggregant variants of TauRD. Oligomers became most evident in the case of the pro-aggregant mutant TauRDΔK280, as monitored by atomic force microscopy, and the fluorescence lifetime of Alexa-labeled Tau (time-correlated single photon counting (TCSPC)), consistent with its pronounced toxicity in mouse models. In cell models or primary neurons, neither oligomers nor fibrils of TauRD or TauRDΔK280 had a toxic effect, as seen by assays with lactate dehydrogenase and 3-(4,5-dimethylthiazol-2-yl)-2,5-diphenyltetrazolium bromide, respectively. However, oligomers of pro-aggregant TauRDΔK280 specifically caused a loss of spine density in differentiated neurons, indicating a locally restricted impairment of function.

## Teaser

**Background:** Tau oligomers are thought to play key roles in Alzheimer disease.

**Results:** Oligomers of the Tau repeat domain do not cause gross neuronal toxicity but decrease spine density.

**Conclusion:** Synaptic toxicity from extracellular Tau oligomers is local, without general loss of viability.

**Significance:** Extracellular Tau oligomers are not likely to explain Tau-mediated neurodegeneration but may cause functional impairment by spine reduction.

## Introduction

Aggregation and deposition of proteins in different tissues can lead to various diseases, collectively termed amyloidopathies (1). This includes brain diseases such as Alzheimer disease (AD)^4^ and Parkinson disease, characterized by the aggregation of Aβ peptide and Tau protein or α-synuclein (2–4). Soluble prefibrillary protein aggregates or “oligomers,” rather than insoluble aggregated proteins, appear to be the most toxic species responsible for cell dysfunction and death (5). The extraction of soluble oligomers from brain along with the identification of the toxic species is a complex problem, and the pathological roles of these entities are difficult to assess (6). Soluble protein oligomers are highly dynamic (7) and heterogeneous (8, 9) and display poor sensitivity toward amyloid-specific probes (10). Thus, understanding the mechanism of oligomer formation and their structural analysis, as well as assessment of their cellular toxicity, is an important ongoing task in the research on neurodegenerative diseases (6, 11, 12).

Intracellular accumulations of the microtubule-associated protein Tau into neurofibrillary tangles (NFTs)) are pathological hallmarks of several tauopathies including AD (13, 14). Although the load of NFTs correlates with cognitive impairment in AD, it is still a matter of debate whether these filamentous aggregates are neurotoxic (15). Recent reports suggest that soluble prefibrillary Tau may be the most toxic species rather than aggregated Tau (16, 17). Employing inducible mouse models expressing human Tau with aggregation-prone mutations (*e.g.* P301L or ΔK280), memory impairment and neuronal loss are observed even before the formation of NFTs, which is ascribed to prefibrillar aggregates (18, 19). Thus, higher aggregates or NFTs are not the primary cause of neurotoxicity and cognitive dysfunction. In fact, the presence of soluble Tau aggregates preceding NFTs correlates well with memory deficits in transgenic mice (20, 21), indicating that early Tau aggregates or Tau oligomers are potential toxic agents in AD and other tauopathies. Moreover, oligomers of recombinant full-length Tau have been found to elicit learning impairment through the disruption of synaptic and mitochondrial functions (17). In AD brain tissue, granular Tau oligomers have been observed and correlated with the degree of dementia (8).

Being a highly soluble and naturally unstructured protein, Tau does not aggregate spontaneously and needs polyanionic co-factors like heparin or RNA for *in vitro* fibrillization. Although the *in vitro* aggregation mechanism of Tau has been investigated extensively (22), little is known about the nature of early intermediates or oligomeric species and their possible roles in several brain diseases (9, 23, 24). The hexapeptide motifs ^275^VQIINK^280^ and ^306^VQIVYK^311^ at the beginning of repeats R2 and R3 are involved in the nucleation of Tau aggregates and therefore are important sites of oligomer formation (25–27). Tau oligomers also have been obtained by cross-seeding either with α-synuclein or amyloid-β oligomers (28). These oligomers are shown to have β-sheet structure and granular morphology along with cytotoxic effects. Recently, employing photochemical cross-linking techniques, it was shown that Tau dimers, which are the earliest species in the aggregation process, assemble to form larger oligomers. By generating monoclonal antibodies against Tau dimers and higher order oligomeric aggregates, an elevated level of Tau oligomers has been demonstrated in the AD brain (26).

Owing to their potential role in Alzheimer disease, Tau oligomers have drawn widespread attention in recent years. However, a number of questions are still not answered. Examples include (i) monitoring the presence and growth of oligomeric Tau species, (ii) the structural properties of Tau intermediates during aggregation, and (iii) the modes of toxicity of intermediates aggregating inside of cells or released outside of cells. In this report we have focused on the aggregation properties of the repeat domain of Tau because it carries the sequence motifs responsible for aggregation. We compared two constructs of 4-repeat TauRD, the wild-type and a pro-aggregant mutant with a ΔK280 deletion mutation (originally discovered in cases of FTDP (29)), which promotes β-structure (30). Moreover, to investigate the effects of phosphorylation both proteins were also studied in their pseudo-phosphorylated form (K*X*GE), exchanging Ser for Glu in the four K*X*GS motifs that control microtubule affinity and aggregation rate. The aggregation behavior was monitored with several complementary imaging and spectroscopic methods, notably atomic force microscopy (AFM) and time-correlated single-photon counting (TCSPC), which are particularly sensitive to oligomeric states. Phosphorylation strongly retards fibril formation but does not prevent it absolutely; however, it supports an extended phase of the oligomeric state. The toxicity of oligomers to cell models or neuronal cultures was minimal by standard assays (lactate dehydrogenase (LDH) and MTT), but in the case of pro-aggregant TauRDΔK280 the toxicity could be detected in terms of a loss of dendritic spines.

## EXPERIMENTAL PROCEDURES

### 

#### 

##### Protein Preparation

**FIGURE 1.**
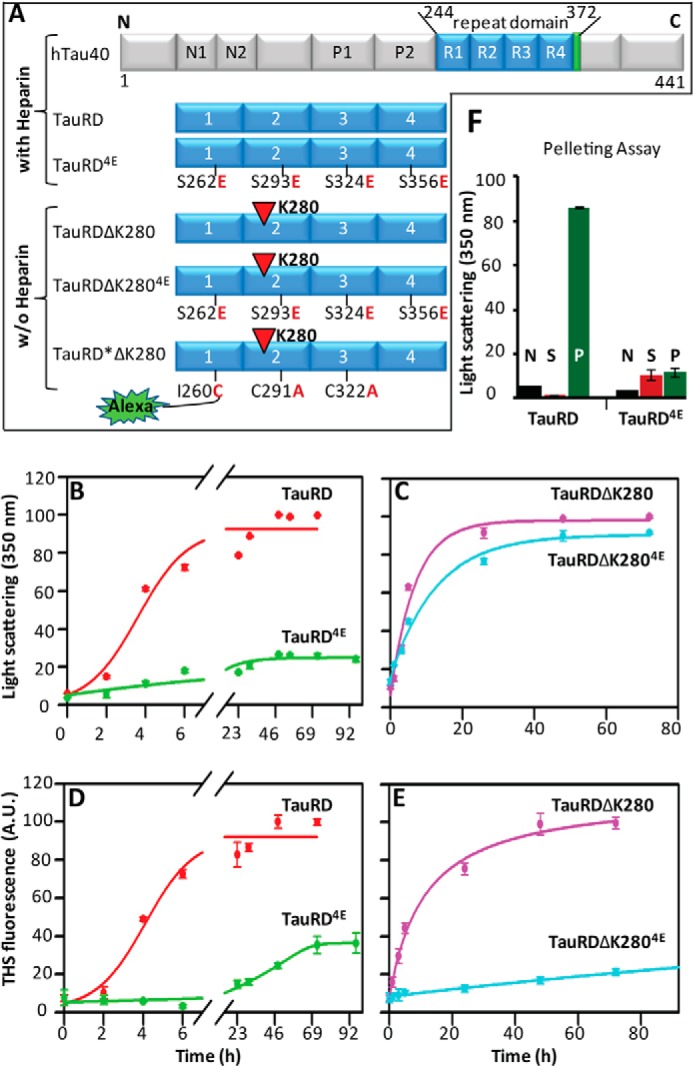
**Aggregation of TauRD variants into oligomers and fibrils and dependence on pseudo-phosphorylation.** TauRD aggregates faster than pseudo-phosphorylated TauRD constructs in the absence of heparin. *A*, overview of Tau constructs used in this study. The full-length hTau40 comprises the 4-repeat domain (amino acids 128–372, highlighted in *blue*), from which TauRD, TauRD^4E^, TauRDΔK280, and TauRDΔK280^4E^ are derived. Fibril formation of TauRD and TauRD^4E^ requires the presence of heparin as a polyanionic nucleating factor, whereas the pro-aggregant deletion mutant at residue Lys-280 (*red triangle*) is able to aggregate without heparin. TauRD^4E^ and TauRDΔK280^4E^ are Tau constructs with pseudo-phosphorylation, where the indicated Ser residues were mutated to Glu (in the K*X*GS motif). For labeling the fluorophore Alexa488 to TauRDΔK280 (see Fig. 4), the native Cys residues 291 and 322 were exchanged for Ala, and a new Cys was introduced by replacing Ile at residue 260 (named TauRD*ΔK280). *B–E*, time course of aggregation monitored by light scattering (350 nm) (*B* and *C*) and by thioflavin S fluorescence (*D* and *E*) in arbitrary units (*A.U.*). TauRD (*red*, *t*_½_ ∼ 5 h) assembles more rapidly by both criteria (*B* and *D*) than pseudo-phosphorylated TauRD^4E^ (4× K*X*GE (*green*)), which increases slowly and only to the level of 20–30%. The scattering intensities (*C*) of pro-aggregant mutant TauRDΔK280 (*magenta*) and its pseudo-phosphorylated construct TauRDΔK280^4E^ (*cyan*) display similar aggregation kinetics (both, *t*_½_ ∼ 6–8 h). However, the ThS fluorescence (*E*) indicates faster aggregation kinetics of TauRDΔK280 (*magenta*) compared with TauRDΔK280^4E^ (*cyan*). Earlier saturation in light scattering intensity (*C*) of TauRDΔK280^4E^ compared with ThS fluorescence (*E*) suggests that the aggregated species are not ThS-sensitive. *F*, monitoring the aggregation of TauRD and TauRD^4E^ after 92 h of incubation (corresponding to final time points in *B* and *D*). Light scattering of particles in supernatant (*S*, *red*) and pellet (*P*, *green*) after centrifugation at 66,000 × *g* and resuspension in PBS buffer. The *black bar* (*N*) represents the scattering intensity of unassembled monomeric protein (starting material, 0 h). Note that TauRD shows pronounced aggregates in the pellet, whereas TauRD^4E^ forms fewer and smaller aggregates/oligomers (*n* = 3 experiments; *error bars* indicate S.D.).

Tau full-length protein (hTau40) and Tau constructs TauRD (also known as K18, residues 244–372, comprising the 4-repeat domain of Tau) and “pro-aggregant” mutant TauRDΔK280 (derived from an FTDP-17 mutation) were prepared as described previously (31) (Fig. 1*A*). Pseudo-phosphorylated variants were generated where the Ser residues in all four K*X*GS motifs were replaced by Glu (4× K*X*GE mutants: TauRD^4E^ and TauRDΔK280^4E^). Tau constructs were obtained in expression vector pNG2 (a derivative of pET-3a (Merck-Novagen), employing site-directed mutagenesis using the QuikChange site-directed mutagenesis method (Stratagene)). Recombinant proteins were expressed in the *Escherichia coli* BL21 (DE3) strain (Merck-Novagen). The expressed proteins were purified from bacterial extracts by using the heat stability of Tau protein and by FPLC SP-Sepharose (GE Healthcare). The cell pellet was resuspended in extraction buffer (50 mm MES, 500 mm NaCl, 1 mm MgSO_4_, 1 mm EGTA, and 5 mm DTT, pH 6.8) supplemented with a protease inhibitor mixture (RocheApplied Science). The cells were disrupted with a French pressure cell and subsequently boiled for 20 min. The soluble extract was isolated by centrifugation, and the supernatant was dialyzed against two changes of cation exchange chromatography buffer A (20 mm MES, 50 mm NaCl, 1 mm MgSO_4_,1 mm EGTA, 2 mm DTT, and 0.1 mm PMSF, pH 6.8) and loaded on a FPLC SP-Sepharose column. The protein was eluted with a linear gradient of cation exchange chromatography buffer B (20 mm MES, 1 m NaCl, 1 mm MgSO_4_, 1 mm EGTA, 2 mm DTT, and 0.1 mm PMSF, pH 6.8). The purity of proteins was ascertained by SDS-PAGE. Where necessary, breakdown products were removed by using the additional gel filtration column Superdex G75 with PBS buffer (137 mm NaCl, 3 mm KCl, 10 mm Na_2_HPO_4_, 2 mm KH_2_PO_4_, and 1 mm DTT, pH 7.4).

##### Labeling of Proteins

For TCSPC experiments TauRDΔK280protein was labeled with Alexa488 dye. In preparation, the native Cys residues 291 and 322 were exchanged for Ala, and a new Cys was induced by replacing Ile at residue 260 (Fig. 1*A*). The aim was to remove the labeled site from the central repeat domain where a bulky dye might interfere with aggregation (32). TauRDΔK280 with those three mutations at residues 260, 291, and 322, is indicated by an asterisk and named TauRD*ΔK280.

Prior to labeling, the protein was incubated in BRB80 buffer (80 mm PIPES, 1 mm MgCl_2_, and 1 mm EGTA, pH 6.8) with a 10-fold molar excess of tris-(2-carboxyethyl)phosphine at room temperature for 30 min for complete reduction of intermolecular disulfide bonds. Thereafter, a 4-fold molar excess of Alexa488 maleimide (Invitrogen) dissolved in dimethyl sulfoxide was added to the protein solution, and labeling was allowed to proceed at room temperature for 3 h in the dark. The unlabeled fluorophores were separated from the labeled protein solution using a NAP-5 column (GE Healthcare) equilibrated previously with BRB80 buffer. The protein concentration was determined by the BCA method and further confirmed by SDS-PAGE with subsequent Coomassie staining. The concentration of bound dye was determined by the molar extinction coefficient of Alexa488 (ϵ_495_ = 72,000 cm^−1^m^−1^). Typically the labeling efficiency was 80–90%. The fluorescently labeled Tau protein was then flash-frozen and stored at −20 °C until use.

##### Assembly of Tau Oligomers and Fibrils (PHFs)

Aggregation of hTau40, TauRD, and TauRD^4E^ were induced by incubating 50 μm soluble protein in volumes of 300 μl at 37 °C in the assembly buffer PBS, pH 7.4 (137 mm NaCl, 3 mm KCl, 10 mm Na_2_HPO_4_, and 2 mm KH_2_PO_4_, ionic strength ∼160 mm) with 1 mm DTT (33) in the presence of the anionic cofactor heparin 3000 (molar ratio of TauRD to heparin = 4:1). Aggregation of TauRDΔK280, TauRD*ΔK280, and TauRDΔK280^4E^ was carried out similarly except without heparin. The formation of aggregates was monitored by thioflavin S (ThS) fluorescence, and the presence of fibrils was confirmed by electron microscopy as described previously (34).

##### ThS Fluorescence

5 μl of 50 μm TauRD proteins in assembly buffer (see above) were diluted 10-fold to 50 μl with ammonium acetate (NH_4_Ac), pH 7, containing 20 μm ThS (final ratio of protein to ThS = 1:4). The ThS fluorescence was measured in a Tecan spectrofluorometer with an excitation wavelength of 440 nm and an emission wavelength of 521 nm (slit width, 2.5 nm each) in a black 384-well microtiter plate with round wells (Thermo Labsystems). Measurements were carried out at 25 °C, and the background fluorescence was subtracted from respective blanks.

##### ANS Fluorescence

5 μl of 50 μm TauRD proteins in assembly buffer was diluted 10-fold to 50 μl with NH_4_Ac, pH 7, containing 20 μm ANS (final ratio of protein to ANS = 5:20 μm = 1:4). All ANS fluorescence experiments were carried out in a Tecan spectrofluorometer at 25 °C using an excitation wavelength of 375 nm and an emission wavelength of 490 nm (slit width 2.5 nm each) in a 384 well plate (black microtiter 384 plate round well; Thermo Labsystems). The background fluorescence was subtracted from respective blanks.

##### CD Spectroscopy

All measurements were carried out with a Jasco J-810 CD spectrometer (Jasco, Gross-Umstadt, Germany) in a cuvette with a path length of 0.1 cm. The parameters were as follows: scanning speed, 100 nm/min; bandwidth, 0.1 nm; response time, 4 s; and measurement temperature, 20 °C.

##### Light Scattering

To monitor the aggregation of Tau constructs, the scattering of 50 μm protein solutions was measured at 350 nm at a 90° angle in a half-micro cuvette (Hellma Analytics). The buffer solution was measured alone and subtracted from each value.

##### Time-resolved Fluorescence Lifetime by TCSPC

Nanosecond time-resolved fluorescence intensity decay was observed using a FluroLog spectrofluorometer (HORIBA Jobin Yvon) employing the TCSPC method. Samples were excited by a pulsed NanoLED at 490 nm. Emission was detected at 515 nm after passing it through 495-nm band-pass filter. The instrument response function was measured using a scattering reference solution of Ludox® (Sigma-Aldrich). To follow the aggregation kinetics of TauRD*ΔK280 by fluorescence lifetime, 2 μm protein (labeled by Alexa488 at the mutated residue Cys-260), and 48 μm unlabeled protein was incubated in PBS, pH 7.4, in the absence of heparin at 37 °C. Following excitation of the sample, fluorescence intensity decay was collected in 1024 channels with a temporal resolution of 0.219 ns/channel. The peak counts were 15,000 for all experiments. Fluorescence intensity decay analyses and lifetime calculations were performed using DAS6 decay analysis software (HORIBA). The total lifetime value (τ) of the dye in labeled protein during the aggregation process was calculated by a superposition of up to three components, using the equation τ = τ_1_α_1_ + τ_2_α_2_ + τ_3_α_3_, where the fluorescence lifetime components (τ_1_, τ_2_, and τ_3_) and their fractional amplitudes (α_1_, α_2_, and α_3_) were obtained by fitting the fluorescence intensity decays of the dye to the minimum number of exponential terms that produces randomly distributed residuals (smallest error). The calculated decay obtained by deconvolution of the observed instrument response function with the chosen decay model (using estimated decay parameters) was fitted to the observed experimental decay profile using the method of iterative deconvolution (35). Nonlinear least-squares fitting based on the Marquardt method was performed to extract the best value for the decay parameters in the intensity decay model. The goodness of fit was evaluated on the basis of reduced χ^2^ and randomness of residuals. A good fit would have a χ^2^ ∼1 and a poor fit a higher value (examples of good fits are shown in Fig. 4, *A* and *B*).

##### Atomic Force Microscopy

For AFM measurements of aggregation time courses, fresh samples of Tau proteins were diluted in PBS to a final concentration of 3 μm and incubated at room temperature. After 0, 2, 96, 144, 168 and 216 h, 20 μl of Tau incubation mix was adsorbed onto a freshly cleaved mica surface for 15 min. Excess Tau protein was removed by exchanging the buffer five times against imaging buffer (PBS). AFM height images (1 × 1 μm, 512 × 512 pixel) were recorded at randomly selected surface positions in peak force mode applying a contact force of 100 pN, an amplitude of 20 nm, and scan rates of 1–1.2 Hz using a NanoScope V (Digital Instruments, Santa Barbara, CA). AFM images were processed using the flattening function of the NanoScope microscope software.

##### Cell Culture

Cryopreserved human neuroblastoma SH-SY5Y cells, purchased from DSMZ (Braunschweig, Germany), were cultured in DMEM supplemented with 15% FBS and 1% penicillin-streptomycin. Cells were maintained at 37 °C in a humidified atmosphere containing 5% CO_2_ and were passaged after trypsinization with trypsin-EDTA solution (Sigma-Aldrich). Cells from the sixth to tenth passages were used for experiments.

Primary cortical neurons were isolated from embryonic E16 mice and plated on poly-d-lysine-coated (50 μg/ml) glass coverslips for immunofluorescence or on coated plastic wells (24-well plates, Corning) for viability assays at a density of 50,000 cells/well. The plating medium was DMEM (high glucose and glutamine) supplemented with 10% horse serum, 1 mm pyruvic acid, and 1% penicillin/streptomycin. The medium was changed to neurobasal medium ((Invitrogen) supplemented with 1% penicillin/streptomycin and 2% B27 (Invitrogen), with 2 mm glutamine (PAA Laboratories GmbH) added after 2–4 h, and the volume was doubled. After 96 h incubation the cells were treated with 300 nm AraC (Sigma) to reduce glia cell growth.

##### Treatments of Cells with Tau Protein

To investigate the effect of the kinetic aggregation states of TauRDΔK280 on cell viability, protein solutions were diluted into serum-free medium for SH-SY5Y cells or, respectively, into conditioned medium for 21-day-old primary mouse neurons and applied to these cells. Protein solutions of TauRDΔK280 were applied at 1 μm total concentration as “monomers” (freshly prepared), “oligomers” (after 3 h of incubation at 37 °C), or “fibrils” (assembled from monomers for 96 h at 37 °C) on neuronal cells. After a 3-h incubation, LDH and MTT assays were performed. Different protein concentrations (0.1 and 10 μm) of TauRDΔK280 monomers and oligomers, as well as longer incubation times with those protein samples (24, 48, and 72 h), were tested on SH-SY5Y cells. To investigate the effect of different Tau aggregates (formed in the presence of heparin) on cell viability, TauRD (monomer and fibril) and hTau40 (monomer and fibril) were diluted in serum-free medium, and 1 μm final protein concentration was applied to SH-SY5Y cells for 3 h before performing the MTT assay.

##### Cell Viability Assay (MTT)

Cell viability was determined with the MTT assay kit (Roche Diagnostics), which is a reporter for mitochondrial activity. Human neuroblastoma SH-SY5Y cells were grown to 70–80% confluency, trypsinized, and resuspended in the medium. The cells were reseeded into 24-well tissue culture plates (2 × 10^4^ cells/well) and maintained at 37 °C in a 5% CO_2_ environment overnight. Primary cortical neurons were plated as described above. After the indicated incubation times with protein samples at 37 °C in a 5% CO_2_ atmosphere, the MTT reagent was added. Viable SH-SY5Y and primary cortical neurons were quantified by measuring the absorption at 550 nm after solubilization of the formazan crystals. As controls, cell toxicity was induced in SH-SY5Y cells by adding 1 mm H_2_O_2_, and in primary cortical neurons by treatment with 2% Triton X-100 for 3 h.

##### LDH Release Assay of Cell Toxicity

LDH released from damaged cells (due to leakiness of the plasma membrane) was measured with a cytotoxicity detection kit (Roche Diagnostics) as recommended by the manufacturer. Briefly, after 3 h of incubation with a 1 μm final concentration of TauRDΔK280 monomers, oligomers, and fibrils (kinetically defined as above), 100 μl of cell supernatant from each well was transferred to 96-well flat-bottom plates and mixed with 100 μl of detection reagent. After 30 min of incubation in the dark, the absorbance of samples was measured at 490 nm using a Tecan plate reader. Absorbance values (LDH release) of primary cortical neuronal cells in different experimental conditions were normalized against 2% Triton X-100. MTT and LDH assays on primary neurons or SH-SY5Y cells were performed three times in triplicate experiments.

##### Immunofluorescence Staining for Spine Quantification

Cells were grown on coverslips for 21 days and then incubated for 3 h with 1 μm TauRDΔK280 (final concentration) or 10% (v/v) PBS mixed into the conditioned cell cultured medium. TauRDΔK280 protein was either freshly dissolved (monomers) or aggregated at 37 °C and 50 μm protein concentration for 3 h (oligomers) or, respectively, for 72 h (fibrils) in the presence of heparin. After this treatment the medium was removed, and the cells were fixed in 3.7% formaldehyde at 4 °C overnight and then permeabilized for 5 min with 0.5% Triton X-100. Actin staining was performed by incubating the fixed cells for 1 h with phalloidin-rhodamine dye (1:100, Cytoskeleton Inc.), and cell nuclei were stained with Hoechst (Sigma). Spines were visualized using the red channel and settings (Cy3) of the cell observer Axiovert 200M microscope (Zeiss) and a ×63 objective. Spines were counted on dendrites at 30 μm distance from the cell soma and over a length of 20–30 μm.

##### Statistical Analysis

**FIGURE 2.**
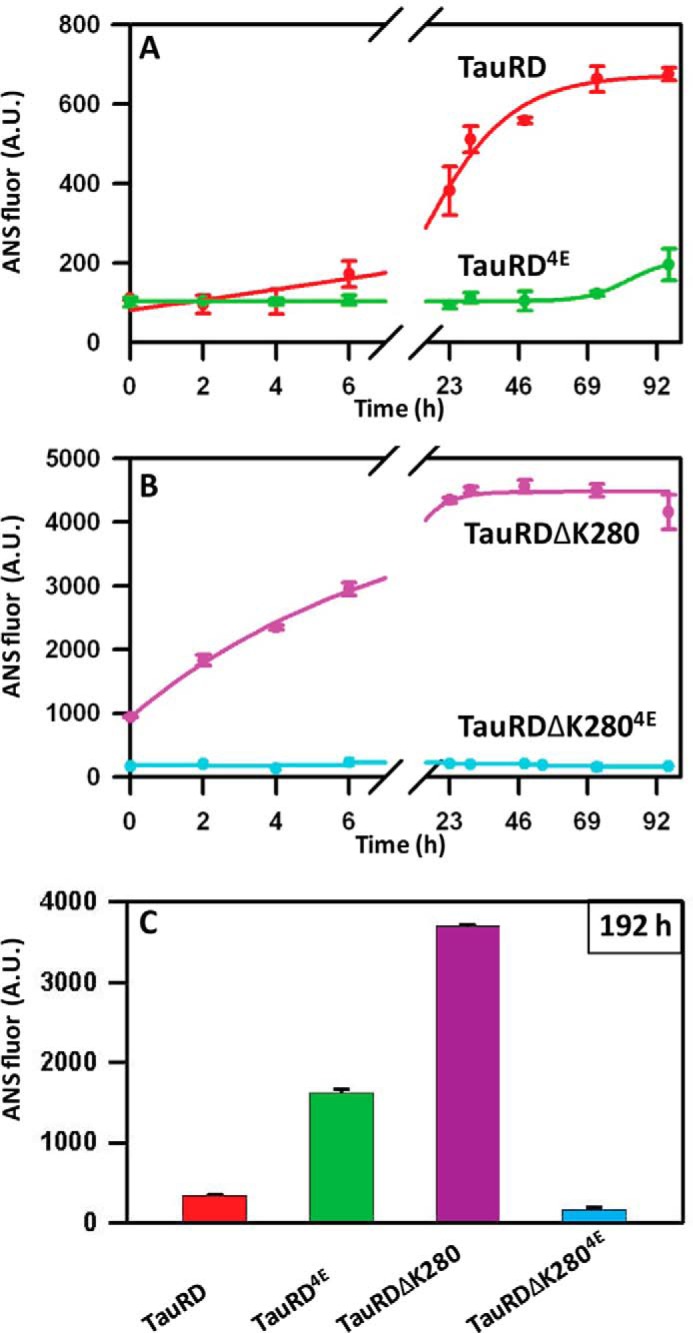
**Conformational changes in different Tau variants during the aggregation process monitored by ANS fluorescence.** The assembly of β-structures is accompanied by ongoing rearrangements of the protein conformation in TauRD proteins. *A*, TauRD shows increased ANS fluorescence after only ∼6 h (*red*, *t*_½_ ∼ 20 h) and saturates after 92 h at a value of ∼650 arbitrary units (*A.U.*). TauRD^4E^ (*green*) shows only ∼28% ANS fluorescence (compared with TauRD) after 96 h. *B*, high ANS fluorescence (∼1000 arbitrary units) from TauRDΔK280 (*magenta*) at the initial time point reflects protein assemblies with altered conformations. The signal increases during the aggregation process and reaches saturation after 30 h at ∼4500 arbitrary units, 7-fold higher than TauRD. On the other hand, TauRDΔK280^4E^ (*cyan*) does not show an ANS signal up to 96 h. *C*, ANS intensities on day 8 (192 h) of incubation. The strongest signal occurs for TauRDΔK280 (*magenta*), indicating that conformational changes occur after the light scattering and ThS signals have saturated (see Fig. 1, *C* and *E*). Both TauRD (*red*) and TauRD^4E^ (*green*) generate less ANS fluorescence. The lowest intensity, and therefore the smallest presence of oligomeric structures, is found for TauRDΔK280^4E^ (*cyan*) at this time point of aggregation. A comparison of the ThS and ANS fluorescence indicates that during aggregation, TauRD first forms amyloid-like structures (visible by ThS), which later undergo a conformational change resulting in increased ANS fluorescence. TauRD^4E^ (*green*) on the other hand, has a long lag phase before aggregation starts (*n* = 3 experiments; *error bars* indicate S.D.).

Data are presented as mean ± S.D. (Figs. 1, 2, and 4*C*) or ± S.E. (Figs. 6 and 7), respectively. Statistical analysis was performed using one-way analysis of variance (ANOVA) followed by Dunnett's post hoc test (**** indicates *p* < 0.0001; *** indicates *p* < 0.001).

## RESULTS

### 

#### 

##### Pseudo-phosphorylation Strongly Retards Aggregation of 4-Repeat TauRD

We first monitored the aggregation of the different forms of 4-repeat Tau (TauRD) (Fig. 1*A*). The Tau constructs TauRD and the pseudo-phosphorylated TauRD^4E^ (4× K*X*GE mutated as indicated) aggregate only in the presence of the polyanionic factor heparin. The term “pro-aggregant” for the construct TauRDΔK280 is derived from the fact that this mutant is able to aggregate slowly even in the absence of polyanionic cofactors such as heparin, in contrast to wild-type Tau (36). Unphosphorylated TauRD and pseudo-phosphorylated TauRD^4E^ (4× K*X*GE) were incubated with heparin 3000 (molecular weight ∼ 3000 Da) at 37 °C, and the extent of aggregation was monitored by static UV light scattering at 350 nm (Fig. 1*B*) and thioflavin S fluorescence (Fig. 1*D*). The aggregation rate of TauRD (*t*_½_ ∼ 4–5 h) is ∼5-fold faster than TauRD^4E^ (*t*_½_ ∼ 20–30 h), which assembles only to a low extent (∼20% of TauRD). Fig. 1, *B* and *D*, illustrates that light scattering and ThS fluorescence develop roughly in parallel, indicating that the assembled structures have a high content of β-structure, corresponding to extended filaments.

A different picture emerges in case of the “pro-aggregant” 4-repeat TauRDΔK280 and its pseudo-phosphorylated form TauRDΔK280^4E^ (Fig. 1, *C* and *E*). The aggregation kinetics of both proteins appear similar by light scattering (*t*_½_ ∼ 4 h, Fig. 1*C*), but the ThS fluorescence (Fig. 1*E*) suggests that TauRDΔK280^4E^ (cyan) is unable to form ThS-sensitive amyloid-like structures. Thus the aggregation process and structural intermediates are different in both proteins. We also quantified the fractions of aggregated TauRD and TauRD^4E^ after 96 h of incubation. The proteins were centrifuged (pelleting at 66,000 × *g* for 35 min), and the resulting supernatants (S) and pellets (P) were analyzed by light scattering (Fig. 1*F*). A comparison of the supernatant and pellet of TauRD revealed a ∼50-fold higher scattering from the pellet (Fig. 1*F*, *green bars*) than from the monomeric protein (*black bars*). On the other hand, the scattering signal from the TauRD^4E^ pellet was 13 times lower and about the same value of the TauRD^4E^ supernatant (Fig. 1*F*, *red bar*). This indicates that TauRD^4E^ aggregated less readily than TauRD. The increased scattering intensity of the TauRD^4E^ supernatant compared with monomeric protein (Fig. 1*F*, *black bars*) indicates the presence of oligomeric particles.

##### ANS Fluorescence Reveals Distinct Conformational Transitions during Tau Aggregation

The dye ANS is a sensitive reporter of protein conformation. A rise in ANS fluorescence is commonly explained by the exposure of hydrophobic microdomains in proteins when folded proteins are unfolded by denaturation (37). Contrary to expectations, the aggregation of Tau is also accompanied by a rise in ANS emission, even though aggregation increases secondary structure, rather than decreasing it, compared with the initial unfolded state (because of partial folding into β-structure). For TauRD the ANS fluorescence rises gradually 2-fold after ∼6 h of incubation and saturates at 7-fold after ∼90 h (Fig. 2*A*, *red*), whereas TauRD^4E^ (*green*) reveals only a small increase (1.5-fold) and only at a later time (>72 h), roughly consistent with the UV light scattering and ThS fluorescence data (Fig. 1, *B* and *D*). Nevertheless, it is notable that the increased ANS fluorescence occurs much later than the saturation of ThS fluorescence, indicating that TauRD and TauRD^4E^ undergo structural rearrangements after aggregation based on β-structure (compare *red curves* in Figs. 1*D* and 2*A*). Fig. 2*B* shows the ANS fluorescence of TauRDΔK280 (*magenta*) and TauRDΔK280^4E^ (*cyan*). In this case the initial intensity of ANS fluorescence of TauRDΔK280 starts at a 10-fold higher level (compared with TauRD, Fig. 2*A*) and then increases further during aggregation, whereas no increase in fluorescence is observed in TauRDΔK280^4E^ even after 92 h of incubation. Together, the data in Figs. 1 and 2 illustrate that the assembly of β-structures is accompanied by continued rearrangement of protein conformation. Note that even after 8 days of incubation there is a significant increase of ANS intensity (Fig. 2*C*) for TauRD^4E^ (*green*), whereas TauRD (*red*), TauRDΔK280 (*magenta*), and TauRDΔK280^4E^ (*cyan*) did not show any further increase. It indicates a significant structural change in TauRD^4E^ after a week-long incubation at 37 °C, possibly related to structural changes within sequence elements contributing to the “fuzzy coat” surrounding the PHF core (38).

##### Changes in Secondary Structure CD Spectroscopy of TauRD during Aggregation

**FIGURE 3.**
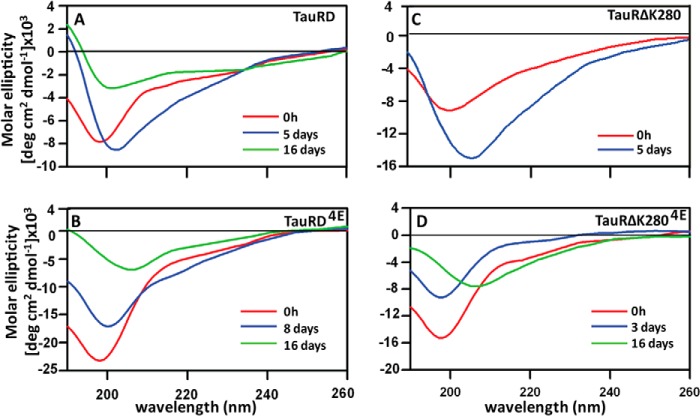
**Time-dependent secondary structures in Tau aggregates.** Pseudo-phosphorylation does not induce pronounced β-structure in TauRD proteins. CD spectra of different Tau variants were obtained by incubating 50 μm protein at 37 °C (in the presence of 12.5 μm heparin (*A* and *B*) and without heparin (*C* and *D*)) and recording 15 spectra at 20 °C. *A*, TauRD shows random coil structure at 0 h (*red*), which shifted toward longer wavelength after 120 h (*blue*) showing little tendency to form β-structure. After 16 days (*green*), the negative peak at 200 nm becomes less pronounced with minima at 198 nm suggesting structural reorganization in Tau molecule. *B*, negative minimum at 197 nm of TauRD^4E^ at 0 h (*red*) changes after 8 days (*blue*) and shifts to a longer wavelength (206 nm) after 16 days (*green*), indicating the formation of some β-structure. *C*, TauRDΔK280 changes from random coil (0 h, *red*) to β-structure after 120 h (*blue*). *D*, TauRDΔK280^4E^ also changes from random coil (0 h, *red*) to increased β-structure after 16 days (*green*). The CD traces from all Tau constructs are initially dominated by a random coil structure.TauRD^4E^ and TauRDΔK280^4E^ aggregates into elevated β-structure after a long incubation period, which is in agreement with the later onset of an increase in the ThS fluorescence compared with the unphosphorylated Tau proteins.

Changes in the secondary structure of different TauRD proteins during aggregation were monitored by CD spectroscopy (Fig. 3). Monomeric TauRD displays a negative trough just below 200 nm, which is typical of natively unfolded or denatured proteins. This is also the dominant feature in all CD traces at the start of aggregation, independently of the conditions and type of protein (Fig. 3, *A–D*, *red curves*). Extended incubation times (Fig. 3, up to 16 days, *green curves*) tend to reduce the depth of the trough, likely caused by increased scattering effects in the samples. The trough also shifts somewhat to the right (between 200 and 210 nm), consistent with an increase in β-structure (27). However, the shift remains small, indicating that the contributions from α- or β-secondary structure remain low (note that a more detailed interpretation of the traces is not realistic because of the heterogeneity of the oligomeric and polymeric species and the scattering caused by the aggregates). Overall the results show that pseudo-phosphorylation does not induce pronounced β-structure in the proteins, even in conditions where oligomerization or fibril formation occurs.

##### Monitoring the Growth of TauRDΔK280 Oligomers and Fibrils by TCSPC

The lifetime of the excited state of a fluorophore is a sensitive parameter for monitoring the microenvironment of a dye. Intrinsic or covalently attached dyes on proteins exhibit a decrease of lifetime during aggregation, typically from several nanoseconds to ∼1 ns or less (39, 40). We monitored the lifetime of Alexa488-conjugated TauRDΔK280 during aggregation by TCSPC. To label the protein via sulfhydryl groups, the native cysteine residues (Cys-291 and Cys-322 within R2 and R3) were replaced by alanine (Ala), and a new single Cys was introduced by a I260C mutation in R1 (termed TauRD*ΔK280). This avoids possible artifacts in fluorescence lifetime due to close apposition of the native cysteines (41) and also avoids interference of the bulky Alexa488 moieties with the aggregation process, as residue 260 is not in the core of the aggregating domain (32). The cysteine alterations in the Tau construct do not interfere with fibril formation (as monitored by electron microscopy and assembly kinetics (42)).

**FIGURE 4.**
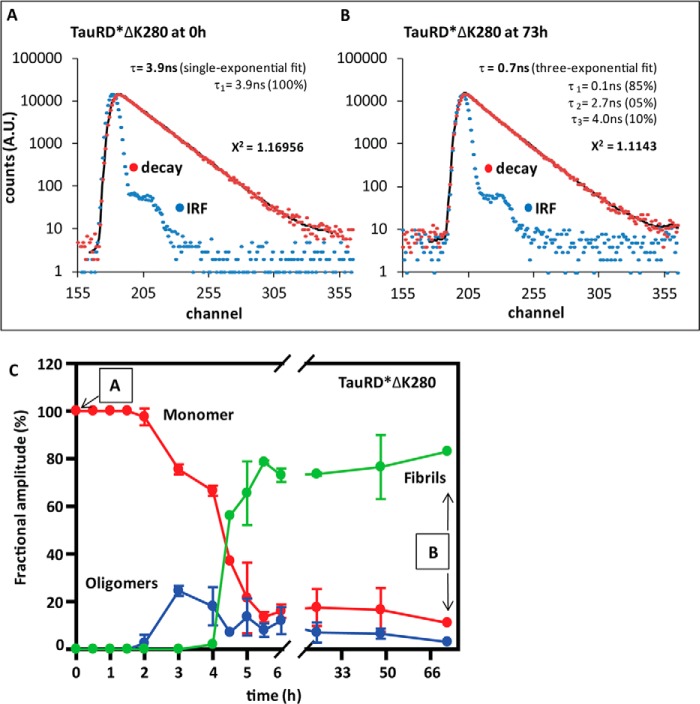
Monitoring the growth of TauRD*ΔK280 oligomers by fluorescence lifetime spectroscopy. *A* and *B*, representative examples of raw data at the beginning (*A*) and the final time point (*B*) of the experiment in *C*. The intensity decay of Alexa488 (*red dots*) and the scattering reference of Ludox® silica (*blue dots*) recorded as the instrument response function is presented. The decay data were fitted (*black line*) aiming for the smallest χ^2^. The data in *A* were fitted by a single exponential fit, as only one lifetime species with an average of 3.9 ns is present. In *B* the best fit was achieved by a three-exponential fit for species with lifetimes of 4.0, 2.7, and 0.1 ns (monomers, oligomer, and polymers, respectively) with fractions of 5, 10, and 85%. The weighted average lifetime is 0.62 ns. The good value of χ^2^ = 1.11 shows that the assumption of three distinct lifetime species is sufficient to explain the data. *A.U.*, arbitrary units. *C*, oligomers of TauRD*ΔK280 appear during the aggregation at 3 h as an intermediate species. Fluorescence lifetime recording was used to monitor time course aggregation of Alexa488-labeled TauRD*ΔK280 in TCSPC mode. The fractional amplitude (a parameter to quantify relative population of species) of monomers (3–4 ns, *red curve*) gradually decreases from 100% to ∼19% after 6 h of incubation during the aggregation process. Oligomers with lifetimes of 1–3 ns (*blue curve*) rise to ∼25% between 3 and 4 h. Fibrils with lifetimes of 0.1–1 ns (*green curve*) appear only after 4 h and reach the saturation level after 6 h. *Error bars* represent S.D.

Furthermore, to ensure that labeled molecules were sensitive to an unlabeled environment in the aggregates, we mixed the labeled protein substoichiometrically (4%, *i.e.* 2 μm labeled A488-TauRD*ΔK280 mixed with 48 μm unlabeled TauRDΔK280). TauRD*ΔK280 has distinct and changing lifetime components during the aggregation process. Fig. 4, *A* and *B*, shows examples of the raw data of the initial (0 h) and final time points (73 h) and the best calculated fit to identify the distribution of lifetime components. The deconvolution of decay data was controlled by residual analysis aiming for the smallest error (χ^2^ ∼ 1). Fig. 4*C* shows the lifetime distribution of Alexa488 in monomeric, oligomeric, and fibrillar TauRD*ΔK280. The monomeric protein displayed a single exponential decay with a lifetime of 4.0 ns, in good agreement with the lifetime of free Alexa488 of 4.1 ns (43). During aggregation, different decay times or lifetime components of Alexa488 were observed, indicating distinct aggregated species. On the basis of measured lifetimes, we grouped the 3–4-ns components as the monomer (Fig. 4*C*, *red*) species of TauRD*ΔK280, 1–3 ns as oligomers (*blue*), and 0.1–1.0 ns as polymers or fibrils (*green*). The binning of lifetime species was based on experimental observations, *e.g.* recordings of aggregation-inhibited TauRD*ΔK280 samples (by incubation at 4 °C) showed a stable lifetime (∼3–4 ns), thus considered as monomers, whereas pelleted aggregated material presented mainly lifetime components below 1 ns.

The amplitude of monomer fraction decreases gradually from 100% to ∼20% after 6 h. Oligomers with 1–3-ns lifetime components were observed only after 2 h of incubation, with a transient peak of ∼25% between 3 and 4 h. After 4 h, polymer (PHF) formation was detected where the fractional amplitude of 0.1- to 1-ns components gradually increased to ∼80% after 6 h. The decrease in the oligomer population with a concomitant increase in fibrils indicates that oligomers are consumed to form the fibrils.

##### Time Course of Transition from Oligomers to Polymers by AFM

**FIGURE 5.**
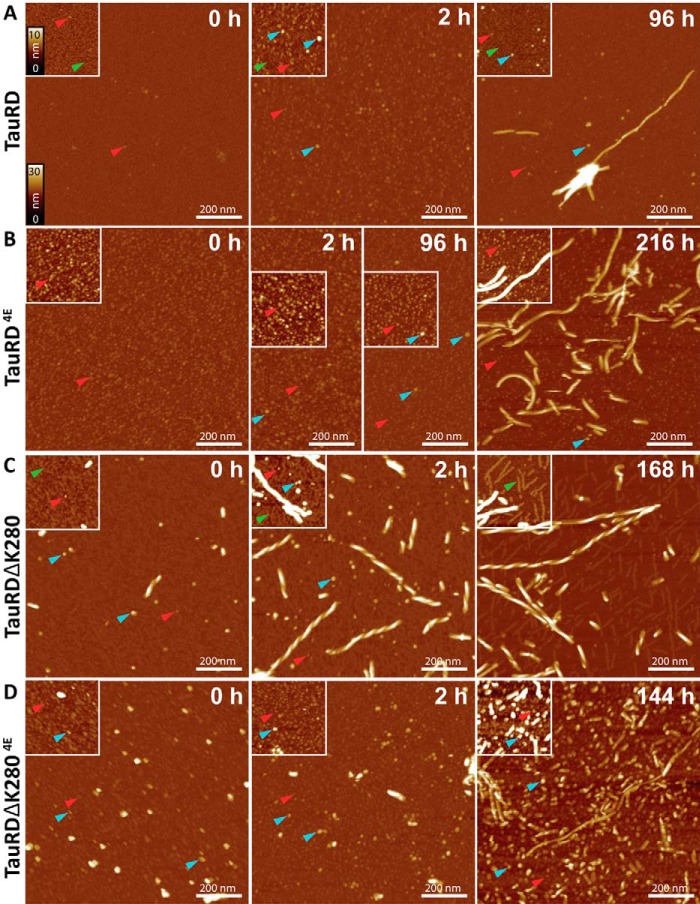
**AFM of aggregation intermediates and fibrils assembled from TauRD.** Monitoring of the aggregation of TauRD proteins with AFM height images recorded at different time points of incubation shows the time-dependent aggregation of TauRD proteins and visualizes small oligomers. Shown are TauRD (*A*), TauRD^4E^ (*B*), TauRDΔK280 (*C*), and TauRDΔK280^4E^ (*D*). One can identify monomers (*green arrows*; maximal height = 1.8 ± 0.3 nm), those growing into oligomers (*red arrows*; maximal height = 3.0 ± 0.4 nm), small aggregates of various maximal heights (*blue arrows*; about 5 nm), and fibrils. *A*, in the starting conditions (0 h) of TauRD, the surface is covered with monomeric TauRD (*green arrow*) and a few small globular oligomers (*red arrows*; maximal height = 3.0 ± 0.4 nm). After 2 h of incubation, a large number of small aggregates (*blue arrows*; maximal height = 3.8 ± 0.7 nm) are present. After 96 h, fewer small aggregates along with mature fibrils are observed. The surface coverage with monomeric TauRD (*green arrow*) at 96 h suggests a persisting excess of monomeric TauRD in solution. *B*, in TauRD^4E^, a large number of oligomers (*red arrows*; height = 3.6 ± 0.6 nm) are observed at the start of incubation (0 h). Only a few small aggregates are present after 2 and 96 h (*blue arrows*; height = 5.0 ± 1.5 nm), and fibrils are seen after 216 h. This is in agreement with ThS and ANS fluorescence, where increased fluorescence of both dyes is observed after 96 h (see Figs. 1 and 2, *green curves*). *C*, AFM topographs of TauRDΔK280 at 0 h show the initial presence of oligomers (*red arrows*; height = 3.2 ± 0.1 nm), small aggregates (*blue arrows*; height = 5.7 ± 2.2 nm), and short twisted fibrils (maximal height = 24.7 ± 0.8 nm). After 2 h, an increased number of oligomers (*blue arrows*) and short twisted fibrils along with mature PHF-like fibrils are observed. After 168 h, long PHFs and short twisted fibrils but few oligomers are seen, suggesting that most oligomers have been incorporated into fibrils. With increasing incubation time, the surface coverage with monomeric TauRDΔK280 (*green arrows*) decreases, and thin protofibrils appear instead. *D*, initially (0 h) in images of TauRDΔK2804E, a large number of oligomers (*red arrows*; height = 3.6 ± 0.4 nm) and different small aggregates (*blue arrows*; heights = 5.7 ± 1.7 nm) are observed. After 2 h, the number of oligomers and short fibrils (maximal height = 24.8 ± 0.3 nm) increases slightly. After 144 h, there is a substantial increase in small aggregates and some “imperfect fibrils.” The *scale bars* in all images are 200 nm. The height scaling from *black* to *white* corresponds to 30 nm in all images and to 10 nm in all *insets*, and the images present randomly chosen surface positions.

In the next step we applied AFM to observe the changes in the morphology of Tau aggregates during assembly. Although electron microscopy revealed the presence of fibrils for all tested TauRD constructs (data not shown; see Ref. 34), we utilized the AFM technique to follow the time course of this aggregation. In the case of TauRD, at 0 h predominantly monomeric protein was recognized (particles of ∼1–2 nm in height) and a few small globular oligomers (maximal height ∼ 3.0 nm) (Fig. 5*A*, *left*). After 2 h of incubation a large number of small aggregates (maximal height ∼ 4 nm) were observed (Fig. 5*A*, *center*), whereas after 96 h, smaller aggregates along with mature fibrils became visible. The morphology of those fibrils appears rather thin and straight in nature. As a rough guide, a sphere of diameter 5 nm could contain ∼4 molecules of TauRD, *i.e.* the dot-like particles contain low-n oligomers, which would not be detected reliably by negative stain electron microscopy.

Fig. 5*B* shows morphologies of TauRD^4E^ assemblies at different time points. A large number of small oligomers with a height of 3.6 ± 0.6 nm were observed at the start of incubation (Fig. 5*B*, *left*, 0 h), but for an extended time, from 2 to 96 h, almost no increase was observed, and only a few small aggregates (maximal height = 5.0 ± 1.5 nm) occurred after 96 h (Fig. 5*B*, *center*). Finally, few fibrils were observed after 216 h (Fig. 5*B*, *right*). The occurrence of fibrils at this time point is in agreement with ThS and ANS fluorescence, where a limited increase in the fluorescence of both dyes was observed after 96 h (Figs. 1, *B* and *D*, and 2*A*). The observation underscores the fact that pseudo-phosphorylation in the repeat domain strongly delays aggregation but does not completely prevent it.

Fig. 5*C* shows different species of the pro-aggregant mutant TauRDΔK280 during aggregation. In this case, oligomers (maximal height = 3.2 ± 0.1 nm), small aggregates (max. height = 5.7 ± 2.2 nm) and short twisted fibrils (maximal height = 24.7 ± 0.8 nm) are already present at the earliest time points (Fig. 5*C*, *left*). This might explain the initial high ANS fluorescence of TauRDΔK280 (Fig. 2*B*). Aggregation proceeded rapidly after 2 h, where numerous oligomers and short twisted fibrils along with mature PHF-like fibrils were observed (Fig. 5*C*, *center*). After 168 h, long PHFs and shorter twisted fibrils but few oligomers were present, suggesting that all oligomers were incorporated into fibrils (Fig. 5*C*, *right*). PHFs formed from TauRDΔK280 in the presence of heparin do not differ in their morphology (data not shown and Ref. 34). They also appear twisted, in a heterogeneous mixture of long and short fibrils, similar to those shown in Fig. 5*C*. The pseudo-phosphorylated mutant TauRDΔK280^4E^ initially reveals large numbers of oligomers (maximal height = 3.6 ± 0.4 nm) and small aggregates (maximal height = 5.7 ± 1.7 nm) (Fig. 5*D*, *left*), but these are strongly inhibited from further growth into filaments. After 2 h oligomers and “amorphous” aggregates (maximal height = 24.8 ± 0.3 nm) were seen (Fig. 5*D*, *center*). The overall appearance of the final aggregates (Fig. 5*D*, *right*) was not different from aggregates formed in the presence of heparin (data not shown). Comparing AFM images (Fig. 5*D*) with the increase in light scattering (Fig. 1*C*, *cyan curve*) but low ThS fluorescence (Fig. 1*E*, *cyan curve*), we concluded that the early species lack β-structure. Even after 144 h, the AFM showed an increase primarily of small aggregates, along with few and imperfect fibrils (Fig. 5*D*, *right*).

##### Effect of Tau Aggregation States on Neuronal Cells

**FIGURE 6.**
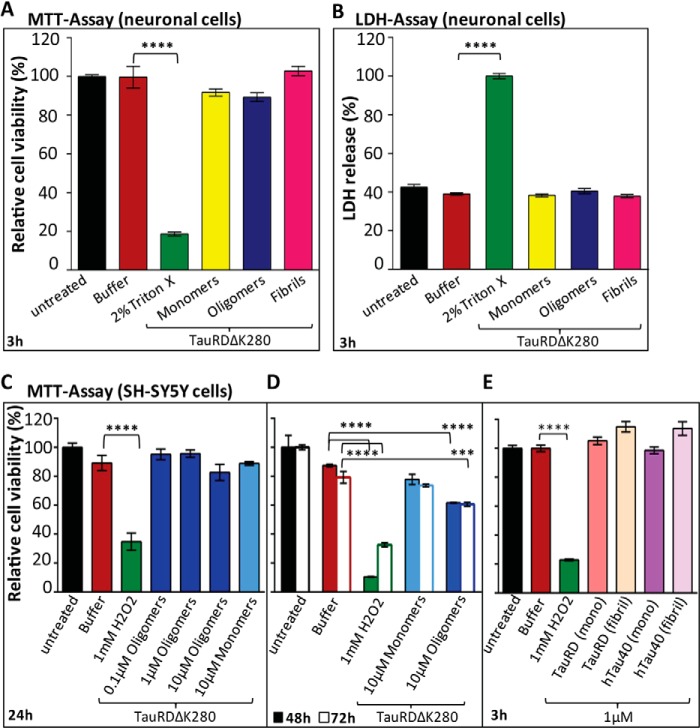
**Effect of TauRDΔK280 aggregates on primary neurons and SH-SY5Y cells.** MTT and LDH assays of mouse primary cortical neurons and SH-SY5Y cells in the presence of TauRDΔK280 oligomers and monomers indicate toxic effects only for long incubation times and high concentrations (10 μm) of TauRDΔK280 oligomers. TauRDΔK280 oligomers were prepared *in vitro* by incubating 50 μm protein for 3 h at 37 °C. Fibrils of TauRD (aggregated for 72 h), TauRDΔK280 (for 96 h), and hTau40 (for 10 days) were prepared according to the conditions described in the legend for Fig. 1*A*, and the fibrils were confirmed by EM. Monomers were added as freshly dissolved protein into the media. *A*, MTT assay. Cells were grown in 24-well plates for 21 days before exposure to TauRDΔK280 samples. After 3 h of treatment with 1 μm monomers, oligomers, or fibrils, cell viability was determined by the MTT assay. *n* = 3 experiments; *error bars* indicate S.E.; ****, *p* < 0.0001 (ANOVA with post hoc Dunnett's multiple comparisons test *versus* buffer). *B*, LDH assay. Cells were grown in 24-well plates for 21 days before exposure to TauRDΔK280 samples. Supernatants of primary neuronal cells incubated for the MTT assay were used for the LDH assay. After 3 h of treatment with 1 μm monomers, oligomers, or fibrils, 100 μl of cell supernatants from each well were taken for the LDH assay. *n* = 6 experiments; *error bars* represent S.E.; ****, *p* < 0.0001 (ANOVA with post hoc Dunnett's multiple comparisons test *versus* buffer). *C–E*, MTT assay, showing treatment of SH-SY5Y cells with different concentrations of TauRDΔK280 (*C*), prolonged incubation with 10 μm concentrated TauRDΔK280 (*D*), and different Tau aggregates at 1 μm concentration (*E*). All values were normalized to the untreated control (100%). *n* = 6 experiments; *error bars* represent S.E.; ****, *p* < 0.0001; ***, *p* < 0.001 (ANOVA with post-hoc Dunnett's multiple comparisons test *versus* buffer). *C*, SH-SY5Y cells were incubated for 24 h with 0.1, 1, and 10 μm TauRDΔK280 oligomers and 10 μm monomers. No significant change in the viability was observed. *D*, SH-SY5Y cells were incubated with 10 μm TauRDΔK280 (monomers and oligomers) for an extended incubation periods of 48 and 72 h. A reduction in viability was observed after cells were exposed to 10 μm TauRDΔK280 oligomers for at least 48 h. *E*, effects of aggregated Tau variants on SH-SY5Y cell viability are monitored by MTT assay. 1 μm TauRD (monomers and fibrils) and the full-length hTau40 (monomers and fibrils) do not show any cytotoxic effect on human neuroblastoma cells after 3 h of incubation.

One aim of this study was to test whether the Tau aggregates could have a toxic effect on cells. Therefore we tested the assembly states of TauRDΔK280 identified by the criteria of fluorescence lifetime analysis (Fig. 4*C*), *i.e.* the fractions of monomers (0 h incubation), polymers (96 h incubation), and oligomers (25% present at 3 h incubation (Fig. 4*C*)). These fractions were applied for 3 h at a total protein concentration of 1 μm to the culture medium of mouse primary neuronal cells followed by MTT (Fig. 6*A*) and LDH assays (Fig. 6*B*). The MTT assay reflects the number of metabolically active viable cells, which did not show a significant decrease in the presence of TauRDΔK280 monomers, oligomers, and fibrils. Another measure of toxicity is the release of LDH into the culture medium by dead cells. Fig. 6*B* shows the LDH release by mouse primary neuronal cells after treating with 1 μm TauRDΔK280 monomers, oligomers, or fibrils. No marked increase in LDH release was observed for any of the Tau species, indicating again that neither of the aggregated fractions is toxic to the mouse primary neurons in terms of gross changes in standard assays.

Next we similarly treated SH-SY5Y human neuroblastoma cells with increasing concentrations of TauRDΔK280 monomers and oligomers for 24 h (Fig. 6*C*) and with 10 μm TauRDΔK280 monomers and oligomers for a prolonged incubation (Fig. 6*D*). Incubating SH-SY5Y cells with the increased concentration of 10 μm TauRDΔK280 oligomers for 24 h did not decrease the viability either (Fig. 6*C*). Only10 μm TauRDΔK280 oligomers, incubated for 48 and 72 h, finally affected the general cell metabolism and led to a significant reduction in MTT (Fig. 6*D*, *blue*). Neither did the same treatment with 10 μm TauRDΔK280 monomers (Fig. 6*D*, *azure*) or buffer (*red*) reduce the viability, although the buffer-treated cells also showed a slightly reduced viability following this prolonged incubation. This enhanced toxicity of oligomers becomes significant only when the cells are incubated for a longer time and possibly weakened by the extended exposure.

To investigate the effect on cell viability of other Tau variants, which aggregate less readily and in the presence of heparin, we treated SH-SY5Y human neuroblastoma cells with aggregated and monomeric full-length hTau40 and TauRD. The neuronal cells were treated for 3 h with 1 μm monomeric protein or fibrils from hTau40 (aggregated for 10 days) and TauRD (aggregated for 72 h). By MTT assay (Fig. 6*E*) we could not observe a significant decrease in cell viability either by full-length Tau (*purple bars*) or TauRD (*pink bars*).

Comparing all of the treatments (Fig. 6), even the efficiently aggregating species TauRDΔK280 did not have a measurable effect, suggesting that extracellular application of Tau, even with enhanced β-structure, does not affect mitochondrial metabolism as seen by the MTT assay. These experiments show that the oligomers have an enhanced toxic potential for cells, as judged by commonly used cytotoxicity and viability assays, but only under rather extreme conditions (Fig. 6*D*).

##### Tau Aggregates Caused a Local Toxicity in Primary Neurons

**FIGURE 7.**
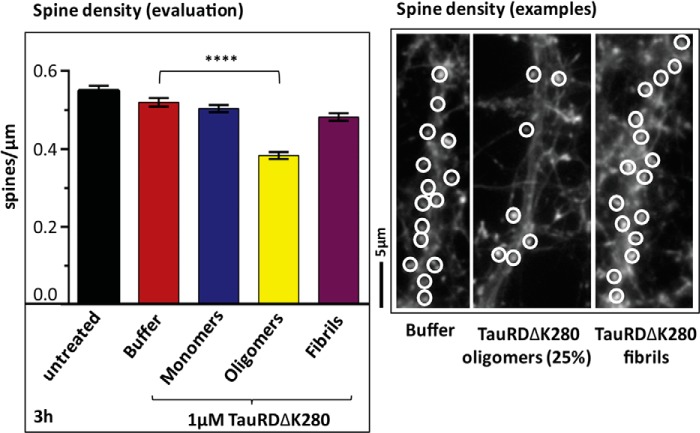
**Effect of TauRD aggregates on primary neurons.** Quantification of spine density on dendrites reveals a localized toxic effect of 1 μm TauRDΔK280 oligomers. This was measured in cultured neuronal cells on the same preparation date as the cells used for the LDH assay described in the legend for Fig. 6*B*. Counting of dendritic spines in primary neuronal cortex cultures was carried out in fixed cells stained with phalloidin-rhodamine as F-actin stain (*highlighting* dendritic spines). At 30 μm distance from the cell soma, a length of 20–30 μm was chosen and the phalloidin-positive spines were counted. Primary neuronal cells (day 21 in culture) were treated for 3 h either with buffer or freshly prepared TauRDΔK280 monomers, oligomers (aggregated for 3 h), and fibrils (aggregated for 72 h). Examples of phalloidin-stained dendrites are shown in the micrographs on the *right*. 30 cells were analyzed for each condition (three different primary cell preparations). The *error bars* represent S.E.; ****, *p* < 0.0001 (ANOVA with post hoc Dunnett's multiple comparisons test *versus* buffer).

These observations prompted us to search for more sensitive indicators that would reflect toxic effects below the level of gross perturbation of metabolism or viability. One such parameter is the density of dendritic spines in mature primary neurons, which reflects the functional state of these cells. Indeed, when exposing neuronal cultures to 1 μm TauRDΔK280 oligomer fraction (Fig. 7, *yellow bar*) for 3 h, there was a significant decrease in spines (34%) compared with only a 9% decrease in the case of 1 μm TauRDΔK280 monomer (*blue*) and fibril fractions (*magenta*). These results demonstrate that TauRDΔK280 oligomers do not grossly alter the membrane integrity or metabolic activity of primary neuronal cortex cells, but they have a more subtle effect that gradually becomes visible in terms of synaptic integrity.

## DISCUSSION

The objective of the present study was to investigate the stages of Tau aggregation into oligomers and fibers using a new combination of methods, to determine the effects of (pseudo-)phosphorylation on these stages, and to test their potential toxicity for cells. The aggregation of Tau is a common feature of Alzheimer disease, frontotemporal dementia, and other “tauopathies” (14). In humans, Tau protein undergoes several posttranslational modifications such as phosphorylation, glycation, truncation, nitration, acetylation, glycosylation, and ubiquitination (44). The phosphorylation of Tau at various sites is an early event in AD pathogenesis. It is thought to reduce the affinity of Tau for microtubules and to promote the formation of neurofibrillary tangles (45).

Pseudo-phosphorylation is a useful way to investigate the effect of site-specific phosphorylation on the structure and function of a protein, and it has been used in multiple *in vitro* and cell culture studies of Tau (46–48). Of particular interest is the repeat domain of Tau because it represents the core of the microtubule binding region and harbors the hexapeptide motifs in R2 and R3 that are responsible for Tau aggregation (3). The repeat domain contains four K*X*GS motifs in which serine phosphorylation by the MAP/microtubule affinity-regulating kinase occurs early in AD. This type of phosphorylation can cause the detachment of Tau from microtubules (49) and the breakdown of the retrograde Tau barrier at the axon initial segment with subsequent missorting (50) but also the inhibition of Tau aggregation (51). Therefore, to clarify the effects of aggregation on a well defined and homogeneous state of Tau, we used pseudo-phosphorylation to study the construct K18, comprising the four repeats of the repeat domain (TauRD and TauRD^4E^), and the pro-aggregant mutants ΔK280 (derived from an FTDP-17 mutation (29)), TauRDΔK280, and TauRDΔK280^4E^. Thus, the phosphomimetic mutations of Ser to Glu were introduced at residues 262, 293, 324, and 356 in repeats R1–R4. Pseudo-phosphorylation in different Tau domains has revealed different effects on its aggregation, depending on the chosen sites and methods of observation (47, 52, 53). Consistent with this, pseudo-phosphorylation in the repeat region (*i.e.* TauRD^4E^) had an inhibitory effect, as observed by both light scattering and ThS fluorescence (Fig. 1, *B* and *D*). The pro-aggregant TauRDΔK280 and its phosphomimetic form TauRDΔK280^4E^ yielded somewhat different results. The low ThS signal indicates that pseudo-phosphorylation inhibits the aggregation of TauRDΔK280^4E^ into β-structure, but UV light scattering reveals that both TauRDΔK280 and TauRDΔK280^4E^ have similar aggregation kinetics (Fig. 1, *C* and *E*). This means that the pro-aggregant ΔK280 mutation counteracts the inhibitory effect of pseudo-phosphorylation and allows assembly, at least to amorphous oligomeric species (see Fig. 5*D*) that lack β-structure. Thus, the two changes can be regarded as mutually antagonistic with regard to aggregation; the ΔK280 mutation pushes the equilibrium toward fiber formation, and the 4E antagonizes this, so that the combined form, TauRDΔK280^4E^, tends to remain in the monomeric or oligomeric non-amyloid form. In structural terms, this can be explained by the results from NMR spectroscopy; the assembly of fibers requires an extended β-conformation around the hexapeptide motifs (54, 55), whereas phosphorylation stabilizes a loop in a non-β-conformation that opposes the stacking of β-strands (56). However, in the monomeric state neither the extended nor the looped structures are stable but undergo pronounced fluctuations. This implies that pseudo-phosphorylation does not inhibit aggregation in an absolute but only a kinetic sense, by delaying oligomerization and fibrillization in TauRD. As a result, even the relatively highly (for its size) phosphorylated TauRDΔK280^4E^ can form some fibers after very long times (Fig. 5*D*).

As mentioned above, the dye ANS is used frequently to probe protein conformation, particularly during unfolding, where an increased ANS signal is thought to reflect the exposure of buried residues of the hydrophobic interior (37). Given this interpretation, it is puzzling to observe an increase in the ANS signal (rather than a decrease) that accompanies the transition from unfolded Tau monomers to semi-ordered aggregates packed into β-structure.

The likely explanation is that ANS does not interact primarily with hydrophobic residues as such but with cationic side chains such as arginine and lysine (57). These residues are numerous in TauRD (21 of 130 residues in construct K18 or ∼15%). Moreover, the fluorescence enhancement of ANS depends on the local structure and is highly selective. For example, in the case of BSA, the ANS signal stems from only 5% of the available cationic side chains (58). Therefore, our preferred interpretation of the results is that some of the Lys/Arg residues increase their affinity for ANS due to conformational changes accompanying aggregation, resulting in an increased fluorescence signal. This can take place even though the major hydrophobic patches of sequence become buried in the aggregate, such as hexapeptide motifs 275–280 and 301–311, which are responsible for aggregation (27). This “inverse” ANS response can therefore be taken as a sensitive marker for Tau conformation in addition to aggregation markers such as ThS.

It is noteworthy that pseudo-phosphorylation strongly suppresses the ANS signal for the pro-aggregant construct TauRDΔK280^4E^ assembled without heparin. In this case, the phosphomimetic mutant does not assemble into β-structured fibers and yet allows precursor aggregates (oligomers; *cf*. Fig. 1, *C* and *E*, *cyan curves*). This argues that the assembly of β-structured fibers is a prerequisite for further conformational changes underlying the ANS signal. Such signals could provide a basis for the further development of PHF-Tau-specific imaging agents (59).

Although Tau aggregates are a hallmark of Alzheimer and several other neurological diseases, it is still a matter of debate whether PHFs are toxic or protective in nature (15). There is growing evidence that prefibrillary protein aggregates (or oligomers) are the most toxic species responsible for cell dysfunction and death rather than highly aggregated proteins (5). The extraction of defined soluble oligomers from brain and other tissues is a difficult task, and so the pathological roles of these entities remain a matter of discussion (24). Additionally, soluble protein oligomers are highly unstable, with a high degree of heterogeneity (60) and poor sensitivity toward amyloid-specific probes.

We therefore investigated the aggregation process of Tau by employing the fluorescence lifetime technique. This allowed us to follow the aggregation dynamics via the change in fluorescence lifetime of a dye attached to the protein. For example, the decrease in lifetime correlates with the appearance of β-sheet-containing amyloid structures (61, 62) or aggregating proteins (63). Monomers, oligomers, and fibrils of Alexa488-conjugated α-synuclein show different lifetimes during the aggregation process (39). In our experiments, the lifetimes of Alexa488 were observed by mixing small amounts of labeled protein with excess unlabeled protein in order to minimize perturbations of the system. In conditions of slow aggregation (*e.g.* TauRDΔK280 without heparin), transient oligomers were noticeable in which the population was highest between 3 and 4 h (^∼^25% (Fig. 4*C*)) before the pattern typical of full-blown fibers emerged. The rise and fall of the oligomer population strongly argues that the oligomers serve as building blocks for the fibers rather than being an off-pathway end state.

Atomic force microscopy has been used extensively to obtain structural information on Tau aggregation (8, 34). The method is particularly suitable for detecting the small and heterogeneous early oligomeric structures that are believed to include the toxic species in several tauopathies and are only poorly visualized by electron microscopy. (As a caveat, we note that not all oligomeric species are necessarily toxic, as shown by the examples of non-toxic off-pathway oligomers of Aβ or Tau (64, 65).)

At the beginning of the assembly process of TauRD, AFM revealed monomeric protein with particles of ∼1–2 nm in height and a few small globular oligomers (height ∼3.0 nm). After 2 h this gave way to large amounts of small aggregates (height ∼4 nm), and after 96 h mature fibrils mixed with smaller aggregates, whereas oligomers became rare again (Fig. 5*A*). This is consistent with the TCSPC data in which oligomers with a 1–3-ns lifetime remained below detectability with TauRD. In the case of the phosphomimetic mutant TauRD^4E^, the fraction of oligomers remains even lower for extended incubation times, but eventually (∼10 days) fibers appear as well. This shows that pseudo-phosphorylation strongly reduces the rate of assembly but does not completely inactivate the protein, so that fibers are formed once nucleation has occurred.

In the case of TauRDΔK280, oligomers are much more dominant, owing to the higher nucleation capacity of this protein (Fig. 5*C*). This correlates with the increased ANS fluorescence early in the aggregation process (Fig. 2*B*) and with the oligomers observed by TCSPC (Fig. 4*C*). After long incubation times most of the oligomers disappeared again, consistent with their role as on-pathway structures, and the sample became populated with fibrillar structures. This kinetic behavior was observed by both TCSPC and AFM, although the time frame of the two methods differs (*i.e.* fibril structures are visible by AFM already at 2 h, before the TCSPC pattern becomes dominated by fibers at 4 h (Figs. 4 and 5)). Here too, pseudo-phosphorylation (TauRDΔK280^4E^) retards the development of fibers but allows the very slow build-up of oligomers (Fig. 5*D*). This may explain the differential effects reported for pseudo-phosphorylated Tau on the aggregation (52, 53).

Perhaps the most pertinent question in the context of Tau aggregation is: what are the possible toxic effects on cells (particularly neurons) and how can a better analysis of Tau aggregation improve our understanding of Tau-dependent toxicity? Several neurodegenerative diseases are linked to Tau aggregation (Alzheimer disease, frontotemporal dementia, Pick disease, and progressive supranuclear palsy), and animal models suggest that some property of Tau protein itself is responsible for the damage to cells. When considering experimental approaches to this issue, one needs to distinguish the toxic effects generated inside and outside of cells. Tau is primarily a cytosolic protein, and therefore several mechanisms have been proposed for Tau-dependent toxicity within cells (*e.g.* loss of microtubule stabilization, interference with intracellular transport or with chaperones, and perturbation of signaling or degradation pathways (66–68).

On the other hand, much attention is currently given to the release of Tau from cells and its subsequent re-entry into other target cells because this might explain the well defined spreading of Tau pathology in AD brains (69–71). Such studies typically focus on the release/uptake process but retain the assumption that Tau toxicity occurs inside of the target cells (17, 72–74). Thus the mechanism of Tau toxicity inside cells remains open, and several mechanisms have been described (75). On the other hand, like many cytosolic components, Tau is also found in the extracellular space at low concentrations, about 45 ng/ml, equivalent to ∼1 nm (depending on the isoform (76)). This is ∼1000 times less than the estimated intracellular concentration in neurons (∼1 μm (77)). How this level of extracellular Tau could be toxic is a matter of debate, but several pathways are conceivable and have been proposed, *e.g.* uptake by cells and propagation of toxic properties (42, 78). Others have recently suggested the modulation of cell surface receptors (79), especially around the synapse (80), or the opening of pores resulting in the leakage of cell membranes (28, 81, 82).

We therefore tested whether the different fractions of Tau characterized here, particularly the repeat domain TauRD and its variants, could induce toxic effects on neurons or cell models as seen by standard assays of cell toxicity. In brief, neither the MTT assay (for metabolic activity) nor the LDH assay (for cell death) revealed significant toxicity at 1 μm pro-aggregant TauRDΔK280 concentration; the same was true for wild-type or full-length hTau40. It required rather extreme conditions, such as high concentrations (10 μm) and extended exposure times, to observe a significant decrease in cell viability in SH-SY5Y cells. In this situation, oligomers were more potent than the monomer or fibril fractions. We conclude that the repeat domain, which is responsible for the aggregation of Tau and which self-assembles more efficiently than full-length Tau, has only a minimal toxic activity when applied extracellularly as judged by standard assays. Toxicity was observed only with more sensitive parameters, notably as a reduction of spine density in primary neurons, and in this case oligomers had a more pronounced effect than monomers or fibers (Fig. 7).

The absence of toxicity by externally added Tau in terms of viability or metabolic impairment is in contrast to other reports. In one study (28) pronounced toxic effects were found for full-length human Tau oligomers on SH-SY5Y cells; after administering 1 μm Tau oligomers obtained by seeding with Aβ peptides, cell viability was reduced by ∼70% as observed by the MTT assay. In another study (81) toxicity induced by 7.5 μm Tau oligomers (in terms of MTT and LDH assays) was ascribed to pore formation in cell membranes. We did not observe these effects, but given the differences noted under “Experimental Procedures” for the characterization of oligomers and measuring their effects, the reasons for the discrepancies are difficult to pin down. Previous experiments have shown that various Tau species can perturb the packing of lipids in mono- and bilayers but do not cause pores (83).^5^ One major difference is that we focused on aggregating Tau species based on the repeat domain with modifications due to phosphorylation or pro-aggregant mutations, because these species are less heterogeneous than full-length Tau assembled with the help of heparin or other cofactors. As shown elsewhere, the expression of the pro-aggregant Tau repeat domain in cell or mouse models is clearly toxic in terms of synapse loss and cell death (19, 84, 85). Therefore, a major conclusion from the present experiments is that extracellular Tau in any assembly state is not likely to cause toxicity (especially at the low concentrations in the interstitial fluid). Conversely, this means that Tau-induced toxicity requires the expression of Tau inside cells or the internalization of extracellular Tau species capable of amplifying toxic effects.

## Footnotes

5 R. Benz and K. Tepper, unpublished observations.

## Footnotes

4 The abbreviations used are:

AD: Alzheimer disease
NFT: neurofibrillary tangle
AFM: atomic force microscopy
ANS: 8-anilino-1-naphthalenesulfonate
FTDP-17: frontotemporal dementia and parkinsonism linked to chromosome 17
LDH: lactate dehydrogenase
MTT: (3-(4,5-dimethylthiazol-2-yl)-2,5-diphenyltetrazolium bromide
PHF: paired helical filament
TCSPC: time-correlated single photon counting
ThS: thioflavin S
ANOVA: analysis of variance.
